# Moderation in Practice and in Statement

**Published:** 1895-03

**Authors:** Safford G. Perry

**Affiliations:** New York City


### International Dental Journal.

Vol. XVI. MARCH, 1895. No. 3.

### Original Communications.[1]

### MODERATION IN PRACTICE AND IN STATEMENT.[2]

BY SAFFORD G. PERRY, D.D.S., NEW YORK CITY.

At the last meeting of this Society, Dr. Jarvie made some very timely remarks on the subject of moderation in practice and moderation in statement of professional experience and opinion.

The lateness of the hour prevented me from expressing approval of his opinions and of adding some thoughts of my own on the same general subject.

Taking his happy expression "moderation" as a text, I would like now, in a rambling sort of way, to express some thoughts which I trust will not be considered too self-assertive, but rather as suggestive to those who care for the real advancement of our calling in all that makes it worthy to be considered a relatively exact and therefore scientific profession.

In the beginning, however, I will say something on the subject of accuracy of observation and expression, and later something on the subject of moderation in practice.

If I commence with a plea for higher scientific attainments, I

[1] The editor and publishers are not responsible for the views of authors of papers published in this department, nor for any claim to novelty, or otherwise, that may be made by them. No papers will be received for this department that have appeared in any other journal published in the country.

[2] Read at a meeting of the New York Odontological Society, November 20, 1894.

shall be only following in the footsteps of many who have talked and written on the subject of dental education and of the means for the elevation of our profession; and I shall expect you to be weary at the outset, for have we not all grown tired of the subject in all its different bearings, and are we not all longing for some new statement of the old story, if we must have any statement of it at all?

If the truth were told, I think we are all unconsciously waiting for some new and more practical way of helping our profession to a higher place, and I venture to suggest that, instead of talking so much about dental education and the training of the young, it would not be out of place if we, who are older, should inspect a little more carefully our own careless methods, and should endeavor to correct and perfect them, so that if we are looked to as examples by the young we may show that, in a new profession in which there must of necessity be much self-education, we have done something, at least, to encourage the scientific spirit, and in doing that, have done the most we can do in our several ways for a profession with which we are so closely linked that we must rise or fall with it as the case may be.

We are weary to the last point of endurance of being told that we are a very scientific body of men, and that our profession is a very exact one. We are tired of being told this, because deep down in our inner consciousness we know it is not true.

In the strict sense of the term we are not very exact in our practice and we are not very exact in the tabulation and report of our experience.

The best proof of this is shown by the fact that in our discussions a statement made, that a certain line of practice is the best known, is often met at once by another statement that the opposite is true.

As both cannot be true, the natural conclusion is that the real truth is somewhere between the two extremes, but we cannot tell just where, and in trying to adjust the sliding scale we finally comprehend the fact that we are not dealing with the exact truth itself at all, but with individual opinions of it, even our own opinion.

Of course there are certain fundamental facts on which our practice rests. For instance, we know the certain action of a few drugs, but we use many more the ultimate action of which we know very little.

We know that under certain conditions certain filling-materials, properly used, have the power to arrest decay of the teeth, but we also know that the same materials, improperly used, or under certain other unfavorable conditions, do not save the teeth for any length of time.

It will be seen, then, that our practice cannot be strictly scientific; but it will be successful in proportion as we apply to it such scientific rules as we are able to master. If we are close observers, and have hand-craft, we may become good practitioners. If we are careless in the observation of facts and hasty in our application of them, we are sure to become uneven and untrustworthy in our practice, even though we may have skilful fingers.

In other words, our personality determines largely the character of our practice.

This is all elementary, and need not be stated, but for the fact that I want to emphasize the importance of what the astronomers call the "personal equation," and to show how largely we are influenced by it in the formation of our opinions, even without suspecting it ourselves.

If, in a science so exact as astronomy, allowance is always made for the personal equation, how much more must it be allowed for in a science so inexact as dentistry is, and must always be.

So exact is astronomy that a slight personal abberation in observing and reporting the stars is quickly detected, but in dentistry, which is so inexact, the wildest personal abberations sometimes go unnoticed and unrebuked.

To eliminate as far as possible the personal equation is a requirement of the first importance, if we are to lead the scientific life.

Confucius insisted upon the acquirement of exact knowledge. He likened imperfect knowledge to a chair with four legs, one of which was broken. "You sit unevenly," he stated, "on such a chair."

Science is concerned with facts, not fancies, and he who can discover the facts and not be influenced by the fancies is the man whose influence will be felt most in the department of science in which he works.

The application of this general proposition to our own profession brings me to the point of repeating that, instead of harping forever about dental education and the elevation of the profession, it seems to me that it would be more becoming and more effective if, after becoming certain of our facts, we adopt the cold, careful, accurate language of science in describing them.

Our literature would then not be so full of wild statements of opinions hysterically expressed.

Exaggeration of statement is a common fault among men and women as we meet them in the world.

Our whole educational system is such, covering, as it does, so vast a range that we are unconsciously careless in our thinking, and naturally we become inaccurate in our habit of statement.

This may be overlooked in the affairs of the world; in fact, there, if people are too accurate, they become a sort of terror, and are called too prim and too precise.

But in the scientific world looseness of thinking and speaking is not permitted, and yet, in our profession it is common because we have not yet thrown off our early habits, and have not yet adopted the accuracy expected of those who lead the scientific life.

The scientific man must also be on his guard against a certain kind of imagination inherent in us all, and which is peculiar to childhood, and not easily outgrown. It is well illustrated by the story of the fisherman, who said the fish he saw swimming towards his hook was a foot long; while pulling him out he thought he must be two feet long, and when he got home and told about it he declared he was three feet long. His unprejudiced friends measured the monster and found him not six inches long. Elated by his day's sport and the champagne of the woods and fields, the fisherman doubtless believed in a misty sort of way that he told the truth.

Men use some new form of gold or amalgam, or gutta-percha or cement, and, charmed by the novelty, they believe and affirm that it is a fish three feet long. And some have the exuberant and hopeful temperament that leads them to think it is a whale!

After a time it shrinks in their estimation and they throw it back and wait for another bite.

The same exaggeration is shown when a runaway horse dashes down the street, and six of the ten spectators say that the carriage-wheels came within two inches of the lamp-post. Careful measurement of the track made by the wheels would probably show that it did not come within two feet of ruin and destruction.

Some men assert and believe that they never have trouble with dead teeth. If their memories could be jogged, and the truth known, it is not unlikely that they have come near having dead patients.

In the same way they assert that in a case of periosteal disturbance they control the inflammation by the use of so and so, which acts upon the circulation in such and such a manner. Aside from the distraction of the nerve-currents caused by the use of counterirritants, what effect could they produce upon the "bugs" swarming around the end of the root of the sore tooth by any remedies applied to the gums?

The Irishman said he could doctor himself, for he put some stuff on his boil and in a week or so it got well.

Words are the tools of the mind. A man with a well-equipped mind is like a carpenter with a good set of tools, which he takes with him, and with which he can work at any time and in any place.

If he is a poet, and is being consumed by some internal canker, his words will be a reflex of his inward pain, and the artist's brush, when he paints, will make a streak of lurid red.

If he is a philosopher, or thinks he is, his words will be ponderous and elephantine and soporific, even to the point of producing a condition, to use his own phrase, of innocuous desuetude, and his Latin terms will be bigger and longer than the things he describes; and his listeners will gape with wonder or fatigue, as the case may be.

If he is a crank, he will be like a hand-organ, which, when wound up, always plays the same tune.

But if he has the scientific training and spirit, his words will be few but to the point. He will say exactly what he means and mean exactly what he says.

My allusion to the carpenter doubtless made you think of the term as applied to the botch dentist. I think it can equally well be applied to some men who are extremely skilful. Their fingers are so nimble, their touch so accurate, and their tools so good that they are forever carpentering away at the teeth, showing what can be done, and how well they can do it. They seek unusual operations, and perform them with a great blowing of trumpets and beating of drums. They exaggerate the importance of their handicraft, and confine themselves within such a narrow range that they lose the larger, wider view, and fail to comprehend the significance of proper dental care, considered in its best and widest sense.

For the tooth-carpenter I would substitute the dental "nurse." You smile at this, as it may suggest to you a society of old grannies, with clumsy hands and feeble brains, but I intend no joke. It is a term I have never seen or heard, but to my mind it expresses an idea that probably will not be appreciated except by those who have had long experience, and have stood by and seen the teeth erupt and develop, and seen them decay and loosen, and have come to know that in the human mouth natural laws that cannot be set aside are at work, and that about all that can be done after all is to nurse and help and conserve.

I recently picked out from the gum a loose molar top-heavy, with a three-hour gold filling that had been put in only three or four years ago, in a beautiful manner by an "operator."

An oxyphosphate filling matched to the color of the tooth, and put in in ten minutes, and renewed three times at the most, with five minutes for each renewal, and a trifling cost for the whole, would have given as good results, so far as usefulness was concerned, and a better result in appearance.

A dental "nurse," with a level head, would have recognized the condition of the mouth, and would have known at once that the tooth could not be retained but a few years at the most, and would have acted accordingly.

There can be no argument made against the nimble fingers that can produce perfect work in all places when perfect work is really required.

There are some teeth so hard to save that human fingers were never yet equal to the task, and so valuable that time and money should and would not be considered for a moment, if they could be saved.

Then the cap and gown of the "nurse," with all the gentleness and good nature that goes with them, must be put aside, and the armor of the "operator" put on, with all the firmness that must ever accompany it.

In addition to rather careless habits of statement, and of not very level-headed practice, there exists in our profession a certain kind of inattention, on the part of many of our good men, and also with some other equally good men, there exists an unconscious intolerance of all ideas and modes of practice that are not in accordance with those they have adopted. This inattention is so wide-spread that any one who ventures to express an opinion, or to describe a certain line of practice, must do so at the risk of being misunderstood, even though he may use the most exact words in our language.

I have experienced this myself many times, but never more markedly than after the publication, last winter, of a paper on the care of young teeth, for which I advocated the plastics, *under certain conditions*, and received the credit, in some quarters, of believing in them not only for all young teeth, but for all teeth.

I have since looked that paper over and I have failed to see how I could have stated any more clearly my faith in gold for teeth of good quality, under favorable conditions, even in young teeth.

The intolerance of which I speak is a trait of our common humanity, and comes down to us through many generations.

It has been shown most, perhaps, in the religious world, but it exists in our profession, as it does in all professions, and it will continue to do so far beyond our time.

One who does not use the plastics will continue to be impatient with one who does, and one who never uses amalgam will pity or abuse the one who has the courage to admit that he sometimes depends upon it. And so on through the whole list.

It requires courage to face the danger of being misunderstood, and it needs patience to bear the scorn of intolerance, but, believing in plastics, under certain conditions, and publicly advocating them requires more courage still, for it puts a weapon into the hands of those who hate you, or those who are jealous of you. But the man who has convictions and lives up to them, must expect to "get it in the neck," anyhow.

It is worth something, however, to be free, and I honor the man who can use plastics in places where he thinks they are called for, without thought of the effect on his reputation.

Dr. Jarvie's phrase "moderation" exactly expresses the need in the dental profession to-day.

Facts, carefully observed and clearly and tersely stated, have great value to earnest seekers after truth in a field where there must always exist to some extent conjecture and uncertainty.

Opinions of those who have had long experience also have great value, but a man's opinions must not be confounded with his facts.

The facts he reports speak for themselves, and are of value as they are clear and plain, and the reverse, as they become vague and shadowy.

But the value of his opinions will depend upon the combination of mental faculties that make up what we call a level head.

If his opinions are to have value, he must show that he sees things as they are, and not as they seem to be, and he must have the power of justly estimating the importance of his facts, and of putting them all in proper relation to each other.

In other words, he must have "horse sense," which is after all only a high order of common sense.

If he has the true scientific spirit, he will be sincere, and being sincere, he will be poised and serene.

He will become so absorbed with his facts that he will forget himself.

His personality will be blotted out, and that which persists will be his array of facts. They can never be destroyed; if they can be, they are not facts!

Without suspecting it he will become unselfish.

We are generally expected to explore the religious world for examples of unselfishness, but I doubt if we ever yet saw a more perfect example of an unselfish man than one who is a true follower of science in the fullest meaning of that term.

He can have no mental struggles, for wherever science leads he must follow.

As he pushes his way into the unknown, acquiring a knowledge of new facts, and discarding his errors, his life becomes a constant going back upon himself, but he has no regrets, for a single new truth outweighs all his old errors.

If this new truth proves a delusion, it will be equally willingly given up.

If he can give to the world a new fact and get the credit for it, he is happy.

If he does not get the credit for it, he is still happy, because the world has got the fact!

If you say, this is ideal, the reply is, that a man who is full of the love of science is full of the love of truth, and a man who is full of the love of truth has a religion that embodies the most perfect ideal.

If he is a dentist and invents some new device, or discovers some way by which his patients and his fellow-practitioners can be benefited, he is happy whether he gets the credit for it or not.

If he puts in a fine filling, he can be content if it is never seen and admired.

If the patient dies, his first thought will not be, "another show-filling gone!"

On the other hand, if the conditions are such that he has to put in a poor one, or such that he thinks he had better put in a poor one, he will be equally indifferent and undisturbed if the filling is seen and condemned.

His love of science will be so strong that his only concern will be to do the true thing, and the true thing will be the right one, and in doing this he will show that he has the level head before mentioned.

And the chances are that the true and right thing will be the moderate one.

In making the practical application of this principle, he will be reminded of his boyhood's copy-book, in which he wrote, "Moderation in all things."

In dental practice he will not use gold exclusively; on the other hand, he will not assert without a qualifying phrase that it is a failure.

He will not use the plastics without regard to the age of the patient—first and second childhood—and the condition of the teeth, and as he grows old himself he will be on his guard against them because they are easy to use.

This danger is an insidious one, and may creep on an elderly man as stealthily as the years have done. We have all doubtless heard gutta-percha called the "old man's filling-material."

There is great danger from plastics, because they are so easily used, and particularly with men who have to submit to the tyranny of large practices, and who have to use them constantly for temporary purposes.

They do so well that it is easy to slip into the habit of using them more generally, and now and then the pendulum quite unconsciously may swing too far the other way.

If moderation were observed it would not swing at all, but would hang balanced between the two extremes.

If our imaginary dentist is young, but desires to be wise, he will be on his guard against the universal over-confidence of youth.

Though the vigor in his blood may make the unattainable seem easy, his mind will be full of interrogation-points, and an introspective habit will save him from making endless mistakes.

He will be careful about the extraction of the sixth-year molars, because some "big-wig" has said they must come out, and he will be slow in undertaking the regulation of teeth that in a few years will regulate themselves.

He will not get frightened out of his wits and lose heart because the teeth of some young patients are soft and seem to be on the verge of destruction, but he will brace up and plaster them up in the best way possible, knowing that a few years will work wonders in the improvement of their condition. He will not lie awake at night and worry about articulation, and decide to build up or down with gold teeth that will be articulated sufficiently for all practical purposes in nature's own way in a few years.

He will remember that millions of men whose teeth were badly articulated have lived long and useful lives; and millions more, in their day and generation, have fulfilled their mission without any teeth at all in their old age! Even George Washington was toothless, and who will say that he lived in vain?

I say that he was toothless, for who that ever saw the numberless masses of carved bone that he is said to have worn would consider them teeth?

I suppose it must be believed that he did wear them all, such as they were, for he could not tell a lie about them; and if *he* could not and did not, it is only reasonable to think that no one else would care to.

I am not aware that history has ever told us why he could not tell a lie.

Perhaps it was because his articulation was so bad. I think these carved bones that we see in museums, as well as in the hands of private collectors, might afford an important clew to the historian. There must have been some such natural impediment, for without it it is hard to believe that a man of his force of character could not tell a lie.

Our young professional friend, desiring to be wise early, will be very slow to condemn as bad practice that which does not conform to his own opinions, and he will not be very ready to say that a certain piece of work was done badly, when he knows little or nothing of the conditions under which it was done.

He will be fortunate if he has the faculty, which is rare indeed, of watching for and profiting by the experience of those who have gone over the course before him.

Wisdom cannot be put on and off as one's coat. It is a slow growth. It has been truly said that by the time men are old enough to know how to live they have to die.

By the time a man has seen a set of teeth develop from early childhood to adult life, and has noted all the changes that occur during that time, he may be ready to practise dentistry wisely in its widest sense, though by that time he may have lost the pliant body that made the performance of difficult operations in cramped positions comparatively easy.

It has often been said of the women who have played Juliet that by the time they were old enough to comprehend the deep significance of the character they were too old to invest it with that sweet quality of girlhood that is always its greatest charm.

But age and experience, even, may not always bring wisdom, for there may be lacking that combination of faculties that makes up what I have called the level head.

This imaginary dentist of whom I speak will, without regard to his age at this time in our profession, find ample need in various ways for the exercise of that moderation of which Dr. Jarvie speaks.

He will be in danger of being led away by the vagaries of bridge-work.

He will find many of his professional brethren gone mad upon that subject, and, caught by the current, he may be swept along and carried out to sea, where, tossing about on the uncertain waters, he may comprehend what good, solid land he has drifted away from.

He may grind down and partially destroy good natural teeth, in order to put in one or two artificial ones which cannot be removed and cannot be kept clean, and which the patient for years had gone without, and yet had not been made specially conscious of their loss.

He may forget that before he was born teeth attached to skeleton plates were put in and held firmly and comfortably, being attached by clasps in such a way as not to injure the natural teeth, and yet so as to allow removal for cleansing or repairs.

He may not perceive that the natural teeth rest in a cushioned socket that allows a little play, and that when his immovable bridge is cemented fast the teeth are held in an immovable manner, nowhere duplicated by nature.

The bridge may eventually come out, but when it does the teeth may come out with it.

If this is considered an extreme statement, my reply is, that I have seen it occur so many times that I consider it should be taken into account as an important factor.

I have taken out a great many immovable bridges; though I have never yet taken one out without being first requested by the patient to do so, for I try to avoid prejudice against this, as against all other special lines of practice. There is no intention in this to condemn removable bridge-work. That is only a modified and greatly improved form of applying an old-fashioned skeleton-plate fastened with clasps. In the matter of gold crowns our professional artist may follow the fashion of the day, and use them so near the front of the mouth that his patient's speech will be gold, and even his silence golden.

Perhaps it is a good advertisement, and leads some people to say, "Who is your dentist?" He may have strabismus, as he evidently does not see that many people ask "Who is your dentist?" in order to avoid him.

The streets, the cars, the churches, and the theatres are so full of people with these glistening gold crowns that it should make one who really cares for the æsthetic side of his profession to hide his head in shame and sorrow.

In the early days the jewellers contributed to our ranks. In these later ones it sometimes seems as if we had gone around the circle only to come back to whence we came.

The setting of diamonds in the teeth is further evidence that some have not lost their early art.

Having eyes, will we never see, and pretending to some comprehension of æsthetics, will we continue to ignore the universally accepted idea, that art that conceals art is the highest; and must we go on inserting in the very mouths of our patients those glistening structures that so completely give them and us away?

Gold crowns placed upon the roots of teeth, as one would place a thimble upon the finger, make the strongest and most durable crowns known to our profession; but our patients are not barbarians, struggling for existence and living in the woods on roots and nuts, that require strong teeth for their mastication.

Instead of that, they are civilized beings,—which means that they have acquired pride of appearance, and have their hair cut, and wear clothes, and try to hide from each other all the physical defects that are the outcome of that very civilization.

And yet it is common for dentists to publish the fact that their patients have poor teeth, by placing gold crowns upon their bicuspids; and once I saw in the mouth of a lady a gold crown placed on a canine, and put there by a man who has a national reputation.

A modern successful novelist carries through one of his books a man whom he describes as a shining example of American dentistry.

To see a glistening gold crown in the mouth of a person is like seeing, as one does in so many hotels in Europe, the sign, "American dentist."

The sight of either gives a peculiar sensation near the diaphragm, and makes one wonder why it is that such a beneficent art can be so paraded and so degraded.

The sane, moderate man does not do these things. His mental make-up is such that he cannot.

If he could be convinced that he ought, he still will not, for he cannot offend his artistic sense even as readily as he can his common sense. Emerson said modern civilization had given the world dentistry and gum shoes. He did not say which he considered the greater blessing. He probably never saw a gold tooth.

We are so tickled with the things we can do by the help of all these modern appliances that we go on doing things that we shall look back upon with wonder and regret when our profession shall have advanced to a fuller appreciation of its true mission, which Oliver Wendel Holmes said was, "to retain the appearance of youth, and to smoothe the wrinkles out of old age." But gold crowns were not known when he wrote that sentence.

There is not time in a paper like this to tell half the things a moderate man will do, nor one-quarter of the things he will not do.

To go further down the list would only exasperate your patience, which is surely exhausted. In rather a jocular, and not a very logical manner, I have gone all round Robin Hood's barn in the consideration of the subject of moderation in practice, and in the matter of moderation in statement. I have given a good illustration of the very thing I have preached against. But possibly you may be willing to admit that the subject is one that allows some latitude, and the impromptu manner in which the paper has been prepared will temper a little, I hope, the severity of your criticism. What I have written has been dashed off in an unstudied manner, and with no time for revision, and without any attempt, inconsistent as it may seem, to eliminate the personal equation. I have only one more thought to express. This imaginary dentist, who, I have claimed, should be so scientific, and to whom I have tried to give a level head, must also be moderate, even with his scientific attainments. I have held him up before you as a cold, exact, unruffled, mathematical, scientific chap, well suited for the lecture-room or the debating society.

Having all these attainments, he might still be a poor stick in the operating-room.

An operator should have warm blood in his veins. Appreciating the weakness and the needs of our common humanity, his heart should be worn on his sleeve, where it can be easily pecked at.

It should be full of tenderness for those of any age who need gentle treatment, and it should be warm with love, particularly when little children come to him with the freshness of spring in their faces.

What would you think of him if, when the little souls ask for a story, he should repeat the differential calculus?

He may have been so absorbed with his scientific studies that perhaps it is the only "Mother-Goose" poem he knows.

He can twirl a drill better than he can turn a joke or a pleasant phrase.

He readily explains that it is not the nerve but the dental fibrillæ that he cuts. Does it hurt any less because he is cutting something that has a Latin name?

No, gentlemen, the dentist should be an all-round man. He should have many sides, and "moderation" ought to be stamped on every one of them.

